# Direct and indirect loading of the Ilizarov external fixator: the effect on the interfragmentary movements and compressive loads

**DOI:** 10.1007/s11751-011-0103-6

**Published:** 2011-02-11

**Authors:** Jan Gessmann, Hinnerk Baecker, Birger Jettkant, Gert Muhr, Dominik Seybold

**Affiliations:** 1 Department of Surgery and Traumatology, BG Universitätsklinikum Bergmannsheil, Bürkle-de-la-Camp-Platz 1, 44789 Bochum, Germany; 2 Department of Surgical Research, BG Universitätsklinikum Bergmannsheil, Bürkle-de-la-Camp-Platz 1, 44789 Bochum, Germany

**Keywords:** Ilizarov, External fixator, Weight bearing, Indirect loading, Interfragmentary movement

## Abstract

The amount of weight bearing and the force transmission to the frame have an important influence on the results of treatment with an Ilizarov external fixator. The frame provides beneficial interfragmentary movements and compressive loads at the fracture site through elastic wires. Mobilisation can be achieved by applying a weight-bearing platform at the distal end of the fixator. The effect on the interfragmentary movements and the compressive loads in indirect and direct loading were analysed in this study using a composite tibia bone model. Displacement transducers were attached to measure the interfragmentary movements and to detect relative movements of the bone fragments and movements between the rings. The compressive loads in the osteotomy were measured with loading cells in the defect zone. The weight-bearing platform had a substantial effect on the biomechanical behaviour of the frame. It led to an indirect force transmission through the fixator with respect to the osteotomy, resulting in lower compressive loads, lower interfragmentary movements and higher mechanical stress on the frame.

## Introduction

The mechanical conditions imposed on a fracture by an external fixator significantly influence the rate of fracture healing and the mode by which union occurs [1, 2]. The optimal mechanical environment for fracture healing is still not exactly defined, but axial micromotions and compressive stress at the fracture site are considered beneficial for bone healing [3–5].

**Fig. 1:**
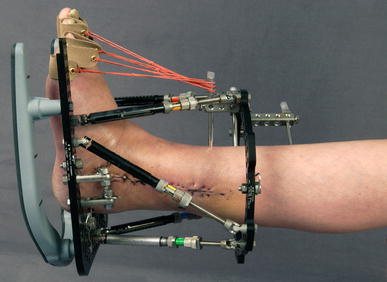
Clinical example of indirect loading with a ring fixator; a weight-bearing platform is attached to the distal ring

The Ilizarov external ring fixator provides a stable yet dynamic system that allows both axial micromotion and compressive loading at the fracture site. The extent of micromotion and the load transferred to the fracture depend on the stability of the Ilizarov frame and on the amount and manner of weight bearing [6, 7]. To gain control of the interfragmentary motions requires an understanding of the various factors affecting the overall characteristics of the fixation device. Various biomechanical studies analysing general construction parameters and configurations that influence Ilizarov frame stability are available [5, 8–13]. One factor that has not been addressed is the mode of force transmission to the frame as a function of the mobilisation of the patient. Usually, patients with an Ilizarov frame are mobilised with direct foot-to-ground contact, which results in equal force transmission from the proximal and the distal bone fragment in the direction of the fracture or osteotomy. However, in cases with a transfixed hind foot, to achieve a stable reduction in a short distal tibial fragment, direct, full weight bearing is not possible. An alternative is a distal extension of the frame with a weight-bearing platform that allows walking with no direct contact of the patient’s foot to the ground (Fig. 1) [14]. This leads to loading of only the proximal tibial fragment on weight loading and to an indirect force transmission from distal extension through the frame into the proximal bone fragment. The aim of this study was to analyse this biomechanical effect on the interfragmentary movements and forces at the fracture/osteotomy site.

## Materials and methods

Direct and indirect loadings were studied on a composite tibia bone model (3rd generation sawbones^®^) with a mid-diaphyseal defect 3.5 mm in size. A universal test machine (UTS^®^, Germany) linked to a multichannel measuring system (MGC-Plus with ML55, HBM^®^) was used for the study. In the set-up for the direct loading, both bone ends were attached to the test machine. For the indirect loading, the struts were distally extended, leaving the distal bone end levitating with no direct contact to the base plate of the test machine (Fig. 3). The attachment to the test machine allowed a solely axial loading parallel to the mechanical axis of the composite bone. The bone was stabilised in a standard Ilizarov frame consisting of 4 × 160-mm rings and four connecting struts (Smith&Nephew^®^, Memphis). Two 1.8-mm wires on each ring were drilled through the tibia in an anatomical position (60° angulation, new wires for every test series), and the tibia was placed more anteriorly in relation to the inner diameter of the ring to more realistically mimic the clinical application. The mid-diaphyseal defect was created with an oscillating saw. At the site of the defect, the distance between the bone and the inner diameter in the anterior-posterior direction was 4.5 cm anterior and 8.0 cm posterior. The wires were tensioned to 1,100 N using the tensioning device that comes with the Ilizarov set and were attached to the rings with slotted bolts.

**Fig. 2:**
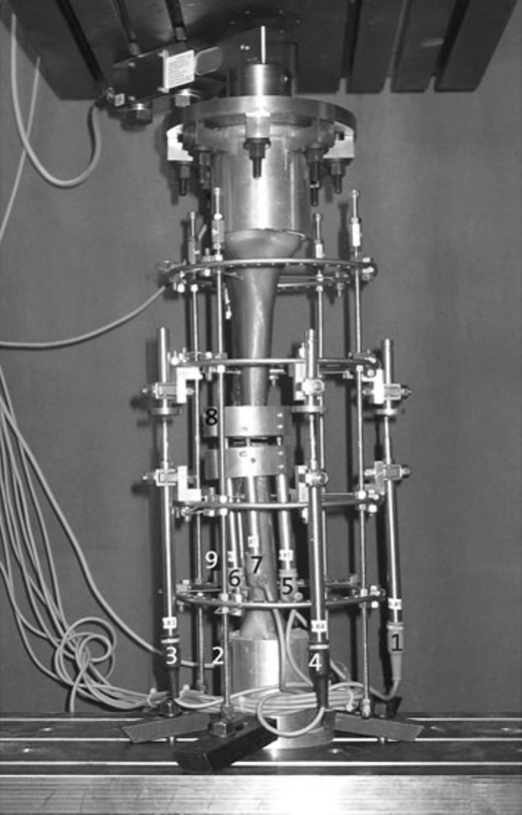
Experimental set-up for direct weight loading; Arrangement of displacement transducers and indicated values: *1*–*4*: Displacement between ring *2* and *3*; *5*–*7*: interfragmentary movements in the osteotomy; *8*–*9*: relative movements of the bone segments in relation to the rings

Inductive standard displacement transducers (WA T, HBM^®^, Germany) were used to measure the interfragmentary motion at the site of the defect, the relative motion of the bone fragments to the rings and the relative motion between the rings. There were three transducers at the site of the defect: two for the relative movements and one for the movements between the rings (see Fig. 2 for the arrangements).

**Fig. 3:**
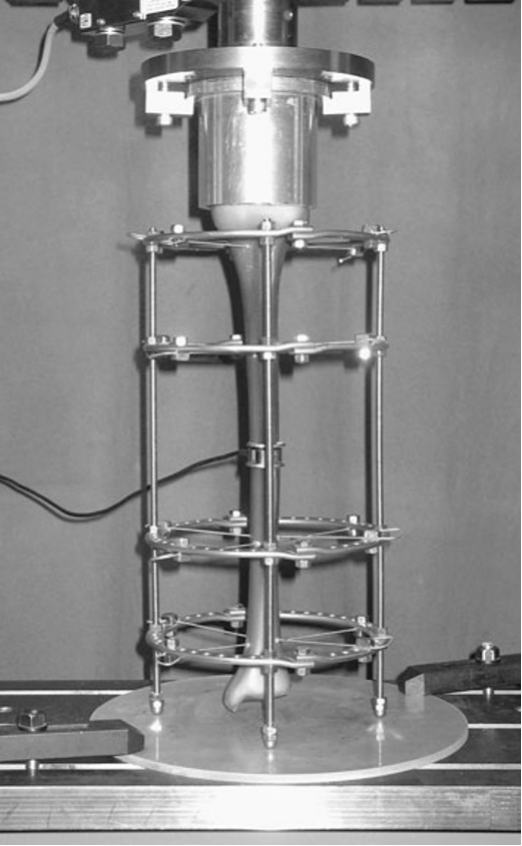
Experimental set-up for analysis of the compressive loads in the osteotomy at indirect weight loading; the connecting rods rest on the base plate, leaving the distal bone end levitating; the loading cell covers the defect completely

In the experimental set-up for the measurement of the forces in the osteotomy gap, a loading cell (FGP Sensors^®^, Fig. 3) was placed in the defect zone.

Continuous axial loading and unloading at a frequency of 5 mm/min was applied to the bone up to 900 N in all tests. To document the reproducibility, each experimental set-up was tested ten times.

## Results

The results from the displacement transducers for the relative movement of the bone showed that at direct weight loading, both bone fragments were pushed towards each other in the direction of the osteotomy. The proximal and the distal fragment covered the same distance in relation to a ring level, which is half of the defect size. Upon contact of both bone ends in the osteotomy, there were no more relative movements of the fragments. At indirect loading, only the proximal fragment moved distally in the direction of the osteotomy and the distal fragment. The proximal segment covered the total defect distance of 3.5 mm. The distal fragment did not move until it was contacted by the proximal bone fragment. Upon contact of the bone ends in the osteotomy, the proximal fragment moved further distally, in the direction of the axial force, and pushed the distal fragment distally. This resulted in greater relative movements of the proximal fragment and therefore greater overall movements.

**Fig. 4:**
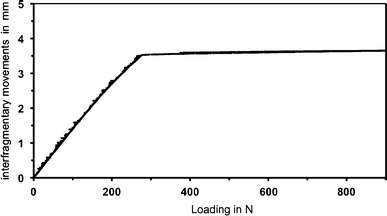
Axial interfragmentary movements at direct loading; averaged results of displacement transducers (*5*–*7*) at the site of the osteotomy; *x*-axis: applied load in N; *y*-axis: interfragmentary movements in mm

**Fig. 5:**
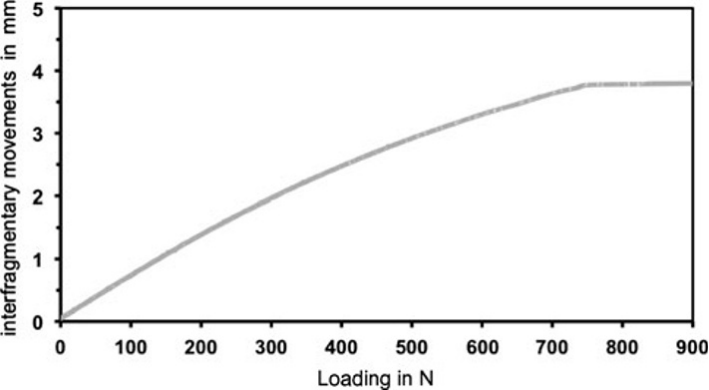
Axial interfragmentary movements at indirect loading; averaged results of displacement transducers (*5*–*7*) at the site of the osteotomy; *x*-axis: applied load in N; *y*-axis: interfragmentary movements in mm

The osteotomy gap closure occurred for direct loading at an axial load of 270 N (SD ± 11) and for indirect loading at an axial load three times higher of 720 N (SD ± 13) (Figs. 4, 5).

No instability of the connecting struts was detected. The displacement transducers showed only very small movements between the rings, with no significant difference between direct and indirect loading. The maximum movement at maximum direct and indirect loading was 0.05 mm.

**Fig. 6:**
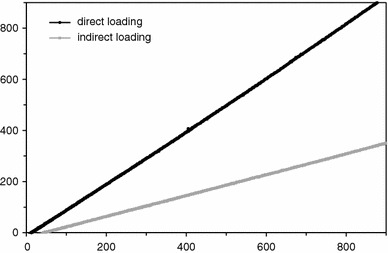
Forces in the osteotomy at direct and indirect axial loading; *x*-axis: applied load in N; *y*-axis: measured force in the osteotomy in N

The loading cell covered the whole defect. This means that the results were measured at an already simulated osteotomy gap closure. At direct loading, there was a linear increase in the measured force in the osteotomy that was directly proportional to the applied axial load. The force increase at indirect loading was also linear to the applied load, but the measured forces were less than half of the values achieved at direct loading. An applied weight load of 500 N, for example, led to a force of 188 N at indirect loading and 500 N at direct loading of the bones (Fig. 6).

## Discussion

The major factors determining the mechanical conditions of a healing fracture or osteotomy, in addition to the biomechanical specifications of the fixation device, the fracture configuration and the accuracy of reduction, are the amount and type of stresses occurring at the bone ends dictated by the functional activity and the loading at the fracture gap [1]. The biomechanical principle of the Ilizarov external fixator relies on axial compressive loads and micromotions that occur on weight loading [4, 5, 11]. Ilizarov and other authors recommend mobilisation of the patients with full weight bearing when treated with the Ilizarov external fixator [15–17], and the beneficial effects have been shown in vivo during distraction osteogenesis [18]. Precise knowledge of the biomechanical effects of the different mounting parts of the Ilizarov fixator is important to estimate the possible amount of interfragmentary motions during treatment. All biomechanical studies on the Ilizarov external fixator were performed with force application on both bone segments as in direct weight loading. This study showed a substantial effect when axial weight was applied only from the proximal bone end, which is the practical effect of a weight-bearing platform.

The results for direct weight loading in this study are consistent with the literature. A slight axial weight load resulted in a relatively large extent of axial movements. Duda et al. [6] detected in vivo interfragmentary movements up to 4 mm in patients treated with an Ilizarov frame and mobilised with direct weight loading and a maximum load of 20 kg. At the same applied load in our experimental set-up, there were axial gap movements of 3 mm. Contact of the bone ends at a plane osteotomy leads to force transmission of all of the axial applied load through the osteotomy and none through the fixator [7]. The loading cell filled the defect fully and therefore simulated an already closed osteotomy gap at the beginning of the measurement of the loading forces in the osteotomy. The forces were directly proportional to the applied axial load, and the load force was one-to-one transferred to the osteotomy.

At indirect loading, the axial gap movements increased at a slower rate in relation to the applied load. To reach the contact point of both bone ends, an axial loading more than 2.5 times higher was necessary. The reason that the needed load was not exactly twice as high as that needed for direct loading might be explained by a self-stiffening effect of the transfixing wires of the proximal bone fragment. Aronson and Harp described an increasing stiffness of the wires with increasing deflection [9]. Because of the increasing stiffness, there is a nonlinearity between the applied load and the wire transverse deflection [19]. The proximal bone fragment in indirect loading covered the whole defect size of 3.5 mm, thereby resulting in a higher deflection of the proximal wires compared to direct loading. The stiffening effect caused by higher deflection of the wires acted as an opposing force and might be the reason for the higher axial loading needed to achieve osteotomy contact in indirect loading.

The forces in the osteotomy in indirect loading reached less than half of the amount of those in direct loading. Strengthening the counter bearing of the distal bone fragment, which in the experimental set-up consisted of the four elastic wires, can be expected to result in higher forces in the osteotomy. A higher stability may be achieved with additional wires or half pins, as demonstrated in particular for axial frame stiffness [8, 10]. Most of the applied axial forces at indirect loading bypassed the osteotomy via the frame instead of being transferred through the osteotomy, which resulted in higher mechanical stress on the frame and wires in general and could lead to higher failure rates from material yielding.

The data presented in this study are from a controlled in vitro model, which leads to limitations in transferring the results to clinical practice. Only an axial load was applied on a plane osteotomy, whereas more complex loading forces interact on an actual bone during weight bearing. Under clinical conditions, increasing the stability of the osteotomy gap will result in changes in the relationship between weight loading and interfragmentary movements as well as in forces in the osteotomy. As there was only air interpositioned in the defect gap, these results need to be considered for the early phase of a treatment with the Ilizarov external fixator when there is no callus formation.

## Conclusion

Application of a weight-bearing platform to an Ilizarov frame that provides better mobilisation results in considerable changes in the biomechanical behaviour concerning interfragmentary movements and osteotomy forces. The distal extension leads to an indirect force transmission through the frame that results in smaller compressive loads and smaller interfragmentary movements in relation to the applied loads, whereas higher mechanical stress remains on the frame.

## References

[1] Aro HT, Chao EY (1993). Bone-healing patterns affected by loading, fracture fragment stability, fracture type, and fracture site compression. Clin Orthop Relat Res.

[2] Yang L, Nayagam S, Saleh M (2003). Stiffness characteristics and inter-fragmentary displacements with different hybrid external fixators. Clin Biomech (Bristol, Avon).

[3] Claes LE, Wilke HJ, Augat P, Rubenacker S, Margevicius KJ (1995). Effect of dynamization on gap healing of diaphyseal fractures under external fixation. Clin Biomech (Bristol, Avon).

[4] Goodship AE, Kenwright J (1985). The influence of induced micromovement upon the healing of experimental tibial fractures. J Bone Joint Surg Br.

[5] Fleming B, Paley D, Kristiansen T, Pope M (1989). A biomechanical analysis of the ilizarov external fixator. Clin Orthop Relat Res.

[6] Duda GN, Sporrer S, Sollmann M, Hoffmann JE, Kassi JP, Khodadadyan C, Raschke M (2003). Interfragmentary movements in the early phase of healing in distraction and correction osteotomies stabilized with ring fixators. Langenbecks Arch Surg.

[7] Paley D, Bianchi Maiocchi A, Aronson J (1991). Biomechanics of the ilizarov external fixator. Operative principles of ilizarov.

[8] Duda GN, Kassi JP, Hoffmann JE, Riedt R, Khodadadyan C, Raschke M (2000). Mechanical behavior of ilizarov ring fixators. Effect of frame parameters on stiffness and consequences for clinical use. Unfallchirurg.

[9] Aronson J, Harp JH (1992). Mechanical considerations in using tensioned wires in a transosseous external fixation system. Clin Orthop Relat Res.

[10] Bronson DG, Samchukov ML, Birch JG, Browne RH, Ashman RB (1998). Stability of external circular fixation: A multi-variable biomechanical analysis. Clin Biomech (Bristol, Avon).

[11] Davidson AW, Mullins M, Goodier D, Barry M (2003). Ilizarov wire tensioning and holding methods: A biomechanical study. Injury.

[12] Mullins MM, Davidson AW, Goodier D, Barry M (2003). The biomechanics of wire fixation in the ilizarov system. Injury.

[13] Osei NA, Bradley BM, Culpan P, Mitchell JB, Barry M, Tanner KE (2006). Relationship between locking-bolt torque and load pre-tension in the ilizarov frame. Injury.

[14] Paley D, Lamm BM, Katsenis D, Bhave A, Herzenberg JE (2006). Treatment of malunion and nonunion at the site of an ankle fusion with the ilizarov apparatus Surgical technique. J Bone Joint Surg Am.

[15] Ilizarov GA (1988). The principles of the ilizarov method. Bull Hosp Jt Dis Orthop Inst.

[16] Shtarker H, David R, Stolero J, Grimberg B, Soudry M (1997). Treatment of open tibial fractures with primary suture and ilizarov fixation. Clin Orthop Relat Res.

[17] McDonald MG, Burgess RC, Bolano LE, Nicholls PJ (1996). Ilizarov treatment of pilon fractures. Clin Orthop Relat Res.

[18] Leung KS, Cheung WH, Yeung HY, Lee KM, Fung KP (2004). Effect of weightbearing on bone formation during distraction osteogenesis. Clin Orthop Relat Res.

[19] Zhang G (2004). Geometric and material nonlinearity in tensioned wires of an external fixator. Clin Biomech (Bristol, Avon).

